# Effects of coronavirus disease 2019 on the incidence, mortality, and prognosis of ischemic stroke: a systematic review and meta-analysis

**DOI:** 10.3389/fneur.2025.1486887

**Published:** 2025-05-13

**Authors:** Xinyue Yang, Wenhao Zhu

**Affiliations:** ^1^First Clinical Medical College, Shandong University of Traditional Chinese Medicine, Jinan, Shandong, China; ^2^Department of Encephalopathy, Zibo Hospital of Traditional Chinese Medicine, Zibo, Shandong, China

**Keywords:** ischemic stroke, COVID-19, incidence, mortality, prognosis, TOAST typing of ischemic stroke, risk factors, systematic review

## Abstract

**Objective:**

The objective of this study was to conduct a systematic review on the effect of coronavirus disease 2019 (COVID-19) on the incidence, mortality, and prognosis of ischemic stroke. The systematic review also ascertained the relationship between COVID-19 and the Trial of Org 10,172 in Acute Stroke Treatment (TOAST) typing of ischemic stroke, as well as the risk factors for ischemic stroke in patients with COVID-19.

**Methods:**

The relevant literature between COVID-19 and ischemic stroke incidence, mortality, and prognosis up to January 2024 were systematically reviewed. Searches were carried out PubMed, Embase, Web of Science, and Cochrane databases. Utilizing the Meta-analysis of observational studies in epidemiology (MOOSE) declaration list, a systematic review and meta-analysis were carried out. Heterogeneity and publication bias were assessed.

**Results:**

Twenty-one studies encompassed 505,864 participants across 13 countries. In total, 1.1% of patients with COVID-19 infection had an ischemic stroke (odds ratio [OR], 0.011; 95% confidence interval [CI], 0.007–0.017; *p* < 0.001). COVID-19 was related to in-hospital mortality (OR, 2.76; 95% CI, 1.90–4.02; *p* < 0.001), mortality 3 months following the beginning of an ischemic stroke (OR, 2.54; 95% CI, 1.80–3.58; *p* < 0.001), and modified Rankin scale (mRS) score ≤2 at hospital discharge (OR, 0.62; 95% CI, 0.54–0.72; *p* < 0.001). mRS ≤ 2 at 3 months after the onset of ischemic stroke did not show any correlation significantly with COVID-19 (OR, 0.67; 95% CI, 0.43–1.06; *p* = 0.086). Longer hospital stays (OR, 0.13; 95% CI, 0.06–0.20; *p* < 0.001) and increased incidence of large-artery atherosclerosis and small-vessel disease phenotypes of ischemic stroke were observed in patients with both COVID-19 and ischemic stroke (*p* < 0.05). In patients with COVID-19, ischemic stroke was substantially linked with hypertension, diabetes, hyperlipidemia, smoking, atrial fibrillation, coronary artery disease, chronic kidney disease, and chronic obstructive pulmonary disease (*p* < 0.05).

**Conclusion:**

COVID-19 is linked with increased incidence and mortality rates for ischemic stroke, as well as a worsening prognosis for the condition. With the data obtained from this study, targeted strategies to prevent and treat ischemic stroke in the context of the COVID-19 can be developed.

**Systematic review registration:**

https://www.crd.york.ac.uk/PROSPERO/view/CRD42024524016, identifier: CRD42024524016.

## 1 Introduction

Stroke is a clinical syndrome resulting from focal or generalized vascular injury to the brain that persists for more than 24 h or leads to death. The second major cause of mortality and the third highest cause of disability, according to the Global Burden of Disease, Injuries, and Risk Factors Study 2017. From 1990 to 2019, the prevalence, mortality and disability rate of stroke have all increased to some extent (1). Therefore, identifying risk factors for stroke and developing reasonable treatment and prevention guidelines are critical. Many diseases can cause stroke, including infections, such as bacterial, fungal, parasitic and a variety of viruses (2). Inflammation increases the risk of stroke by accelerating atherosclerosis, vasculitis, and vasculopathy (3). Infectious diseases triggered by severe acute respiratory coronavirus-2 (SARS-CoV-2) were identified as coronavirus disease 2019 (COVID-19). As it is highly contagious, the disease spread rapidly worldwide after its emergence, sparking a pandemic (4). COVID-19 affects several systems, leading to systemic inflammation and damage to the respiratory and nervous systems (5). Recently, there has been significant attention on the reported link between COVID-19 and ischemic stroke. There is some evidence that COVID-19 may contribute to ischemic strokes (6–8). In addition, there is still a lack of clear evidence-based medical consensus on the impact of reporting on the prognosis, mortality, and morbidity of ischemic stroke, the relationship between COVID-19 and TOAST typing of ischemic stroke, and the impact of COVID-19 on risk factors for ischemic stroke in patients. This review investigated whether COVID-19 affects the incidence, mortality, and prognosis of ischemic stroke.

## 2 Materials and methods

### 2.1 Study search

This study was registered in PROSPERO database (CRD42024524016). We adhered rigorously to the guidelines of the preferred reporting items for the meta-analysis of observational studies in epidemiology (MOOSE) declaration list. When designing and conducting this study. PubMed, Embase, Web of Science, and Cochrane databases were thoroughly searched from inception to January 2024. To identify relevant literature, we searched for predefined keywords (available in Supplementary material). To ensure that the search method was sensitive, the citation lists retrieved were subject to manual screening.

The inclusion criteria were as follows: (i) clinical research articles, such as cohort, case–control, and randomized clinical trials, were considered; (ii) participants were patients diagnosed with ischemic stroke through computed tomography or magnetic resonance imaging, with no comorbidities, serious organic diseases, or complications; and (iii) published literature in English.

The exclusion criteria were as follows: (i) duplicate studies; (ii) animal experiments, reviews, and conference papers; (iii) literature that was not fully accessible; and (iv) literature with incomplete data.

### 2.2 Data abstraction and quality assessment

The eligibility of papers for inclusion was assessed by two researchers separately. If there was a discrepancy in study selection, a third individual was consulted to make the final determination.

The studies were organized based on the type of literature, basic characteristics of the study population, mean, standard deviation, odds ratio (OR), and 95% confidence interval (CI).

Two separate researchers extracted and evaluated the data. The primary author, date published, study source, sample size, study design, study outcomes, and purpose of the meta-analysis were extracted. Upon completion, the work was subjected to a thorough examination for any omissions or errors.

To evaluate the quality of studies, the Newcastle–Ottawa Scale (NOS) was employed. High-quality literature was defined as studies with scores >6 (9).

### 2.3 Statistical methods

Stata MP 14 (StataCorp, College Station, TX) was utilized for statistical analysis. ORs and 95% CIs summarized effect sizes. The chi-squared Q test and *I*^2^ statistics were used to evaluate heterogeneity. *I*^2^ was >50% or *p* value was <0.05 suggested significant heterogeneity, thereby requiring a random-effects model. If not, a fixed-effects model was employed. The visual evaluation of publication bias was performed through Egger’s test and Begg’s funnel plot.

## 3 Results

### 3.1 Study selection

**Figure 1:**
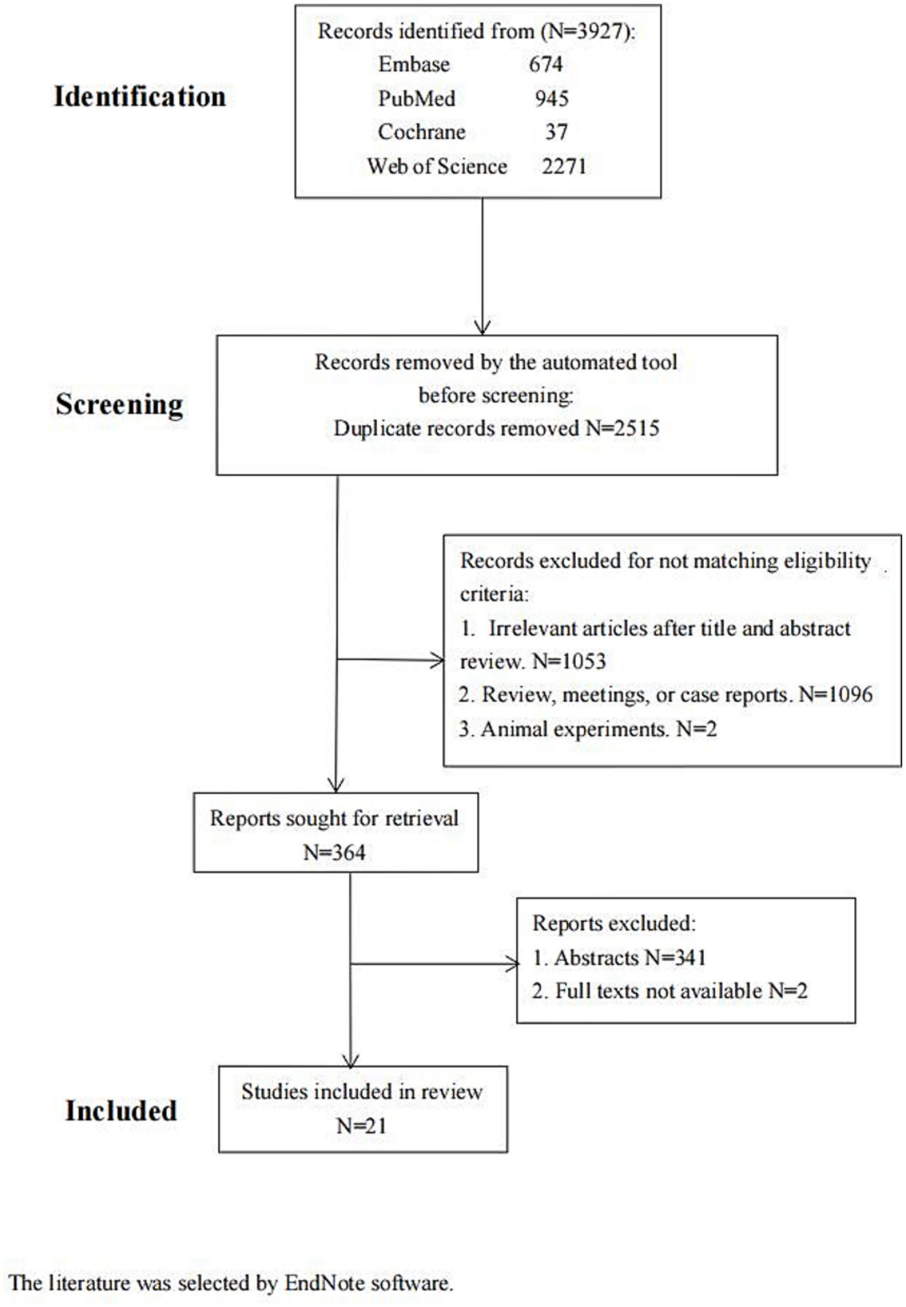
Flow diagram of the study selection process.

There were 3,927 articles found in Embase (*n* = 674), PubMed (*n* = 945), Web of Science (*n* = 2,271), and Cochrane (*n* = 37). Upon examination of the abstracts and titles, 1,053 articles were excluded from the subsequent analysis. Then, 1,096 articles and two animal studies were excluded after a thorough review of the remaining literature in compliance with the established inclusion criteria. Further, 364 articles were screened later. Of these, 341 conference papers and two without full text were discarded. Finally, 21 papers were included (Figure 1; Table 1). The literature was selected by EndNote software.

**Table 1:** Study baselines.

Authors	Country	Study design	Reported outcomes	*N*
Takács et al. (22)	Hungary	Retrospective cohort study	mRS ≤ 2 at discharge; in-hospital mortality; TOAST typing of ischemic stroke; length of hospital stay	83
Yaghi et al. (23)	USA	Retrospective cohort study	In-hospital mortality; TOAST typing of ischemic stroke	78
Qureshi et al. (14)	Colombia	Retrospective cohort study	Incidence; in-hospital mortality; risk factors for ischemic stroke; length of hospital stay	27,676
Srivastava et al. (24)	USA	Retrospective cohort study	Incidence; in-hospital mortality; mRS ≤ 2 at discharge; TOAST typing of ischemic stroke	41,971
Ntaios et al. (62)	USA	Retrospective cohort study	TOAST typing of ischemic stroke	5,858
Martí-Fàbregas et al. (30)	Spain	Prospective observational cohort study	Mortality after 3 months; TOAST typing of ischemic stroke; length of hospital stay	701
Sluis et al. (12)	Netherlands	Retrospective cohort study	Incidence; risk factors for ischemic stroke	2,147
Ramos-Araque et al. (63)	USA, Spain, Egypt, Romania	Retrospective cohort study	In-hospital mortality	14,483
Calmettes et al. (25)	France	Retrospective observational study	mRS ≤ 2 at discharge; in-hospital mortality; TOAST typing of ischemic stroke	216
Strambo et al. (31)	Switzerland	Registry-based study	Mortality after 3 months; TOAST typing of ischemic stroke	2,341
Wang et al. (26)	China	Retrospective cohort study	Incidence; risk factors for ischemic stroke	36,358
Davis et al. (64)	USA	Retrospective cohort study	Incidence	329,240
Lekoubou et al. (65)	USA	Retrospective cohort study	Incidence; risk factors for ischemic stroke	8,815
Al Qawasmeh et al. (10)	Jordan	Prospective cohort study	In-hospital mortality; length of hospital stay; mRS ≤ 2 after 3 months; TOAST typing of ischemic stroke	178
Sierra-Hidalgo et al. (66)	Spain	Retrospective cohort study	Incidence; in-hospital mortality; length of hospital stay	8,126
Merkler et al. (27)	USA	Retrospective cohort study	Incidence; risk factors for ischemic stroke; in-hospital mortality	1916
Ciolli et al. (11)	Italy	Retrospective cohort study	mRS ≤ 2 after 3 months; mortality after 3 months; TOAST typing of ischemic stroke	137
Shakil et al. (13)	USA	Retrospective cohort study	Incidence; risk factors for ischemic stroke; in-hospital mortality	21,073
Elsheshiny et al. (28)	USA	Prospective cohort study	mRS ≤ 2 at discharge; in-hospital mortality; TOAST typing of ischemic stroke	399
Osman et al. (29)	India	Retrospective cohort study	Incidence; mRS ≤ 2 after 3 months; mortality after 3 months; risk factors for ischemic stroke; TOAST typing of ischemic stroke; length of hospital stay	1,369
Cho et al. (67)	USA	Retrospective cohort study	Incidence; risk factors for ischemic stroke	2,699

### 3.2 Study characteristics and quality evaluation

The review comprised 21 studies, of which 20 were categorized as cohort studies and one as an enrollment study. Every analytical study was of excellent quality (NOS ≥ 7; Table 2).

**Table 2:** The Newcastle–Ottawa quality assessment scale.

Author	Selection of the study groups	Group comparison	Outcome	Total score
Takács et al. (22)	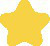	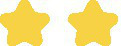		8
Yaghi et al. (23)	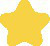	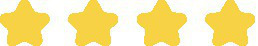		9
Qureshi et al. (14)		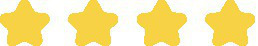		8
Srivastava et al. (24)	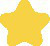	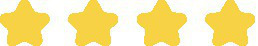		9
Ntaios et al. (62)	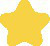	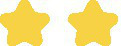		8
Martí-Fàbregas et al. (30)	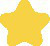	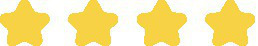		9
Sluis et al. (12)	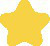	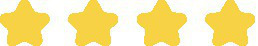		9
Ramos-Araque et al. (63)		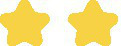		7
Calmettes et al. (25)		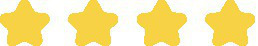		8
Strambo et al. (31)		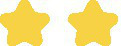		7
Wang et al. (26)	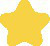	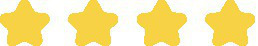		9
Davis et al. (64)	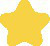	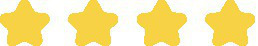		9
Lekoubou et al. (65)	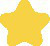	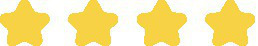		9
Al Qawasmeh et al. (10)		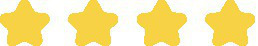		8
Sierra-Hidalgo et al. (66)		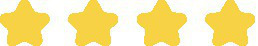		8
Merkler et al. (27)	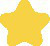	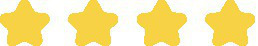		9
Ciolli et al. (11)	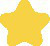	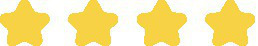		9
Shakil et al. (13)	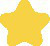	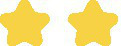		8
Elsheshiny et al. (28)	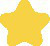	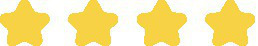		9
Osman et al. (29)		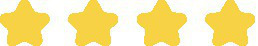		8
Cho et al. (67)	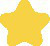	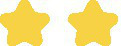		8

### 3.3 Incidence of ischemic stroke in patients with COVID-19

**Figure 2:**
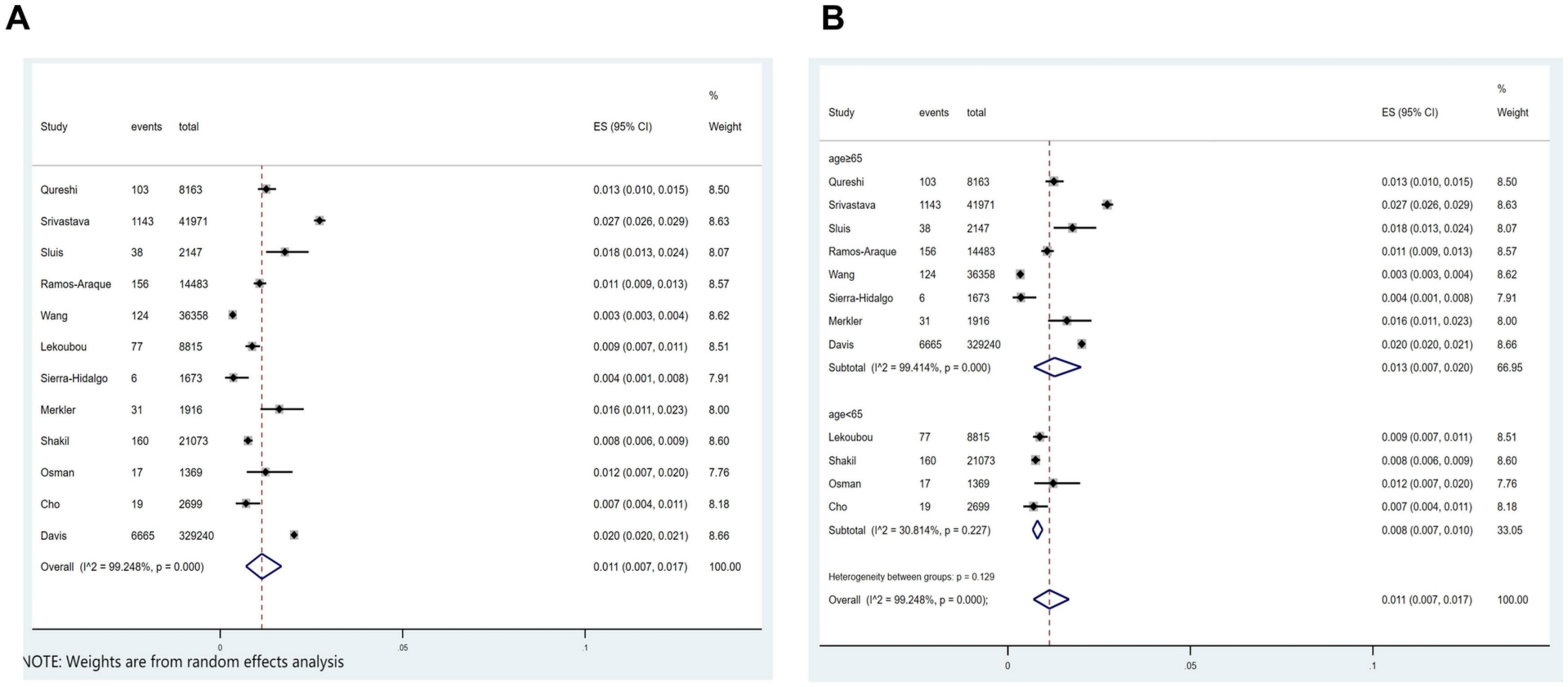
Forest plot illustrating the incidence rate of ischemic stroke in patients with COVID-19 **(A)** and subgroup analyses **(B)**.

Out of the 21 studies, 12 indicated an incidence of COVID-19 combined with ischemic stroke, as illustrated in Figure 2A, with a prevalence rate of 1.1% (*p* < 0.001; *I*^2^ = 99.25%; random-effects model) (OR, 0.011; 95% CI, 0.007–0.017; *p* < 0.001).

Subgroup analysis was performed according to age (age≥65 vs. age<65). The results showed that the incidence of ischemic stroke in COVID-19 patients aged≥65 and aged<65, respectively, was 1.35 and 0.8% (Figure 2B).

### 3.4 Effect of COVID-19 on in-hospital mortality in patients with ischemic stroke

**Figure 3:**
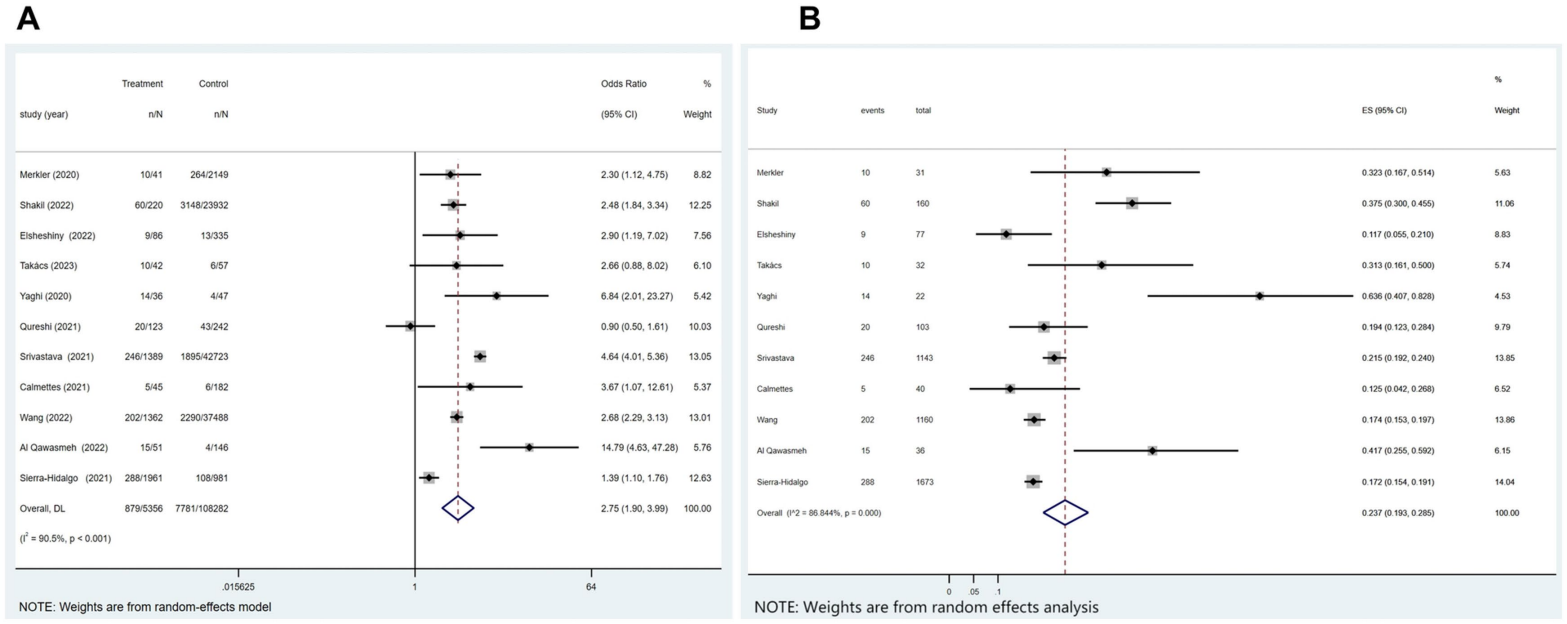
Forest plot illustrating the effect of COVID-19 on in-hospital mortality in patients with ischemic stroke **(A)** and mortality rate **(B)**.

Of the 21 studies, 11 investigated the relationship between COVID-19 and in-hospital mortality associated with ischemic stroke. Figure 3A showed that COVID-19 significantly correlated with ischemic stroke-related in-hospital mortality (*p* < 0.001; *I*^2^ = 90.5%; random-effects model) (OR, 2.75; 95% CI, 1.90–3.99; *p* < 0.001) and Figure 3B illustrated that the mortality rate was 23.7% (OR, 0.237; 95% CI, 0.193–0.285; *p* < 0.001).

### 3.5 Effect of COVID-19 on mortality rate 3 months after the onset of ischemic stroke

**Figure 4:**
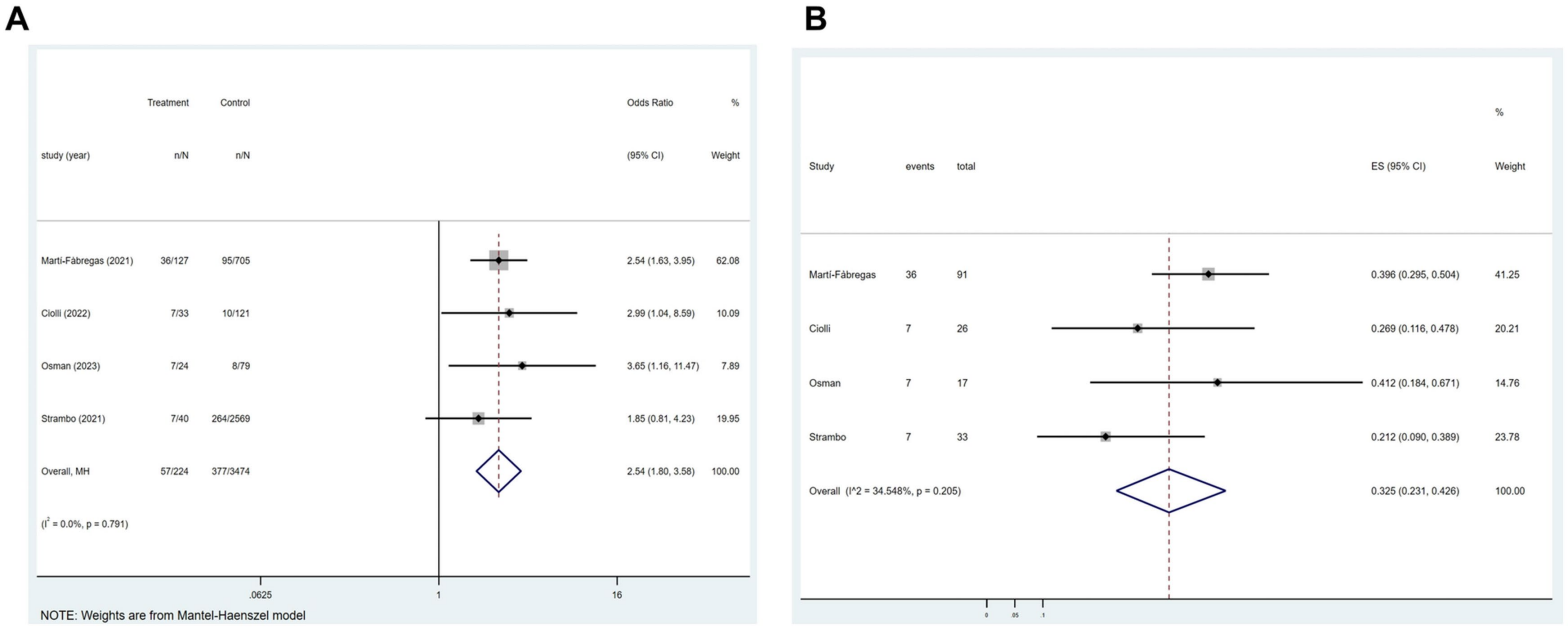
Forest plot illustrating the effect of COVID-19 on mortality rate 3 months after the onset of ischemic stroke in patients with ischemic stroke **(A)** and mortality rate **(B)**.

In four of the 21 studies, the association between COVID-19 and mortality at 3 months after the onset of an ischemic stroke was analyzed. Figure 4A indicated a substantial association between the variables (*p* = 0.791; *I*^2^ = 0.0%; fixed-effects model) (OR, 2.54; 95% CI, 1.80–3.58; *p* < 0.001). Further analysis, as shown in Figure 4B, showed a mortality rate of 32.5% (OR, 0.325; 95% CI, 0.231–0.426; *p* < 0.001).

### 3.6 Relationship between COVID-19 and mRS ≤ 2 at different states in patients with ischemic stroke

#### 3.6.1 At hospital discharge

**Figure 5:**
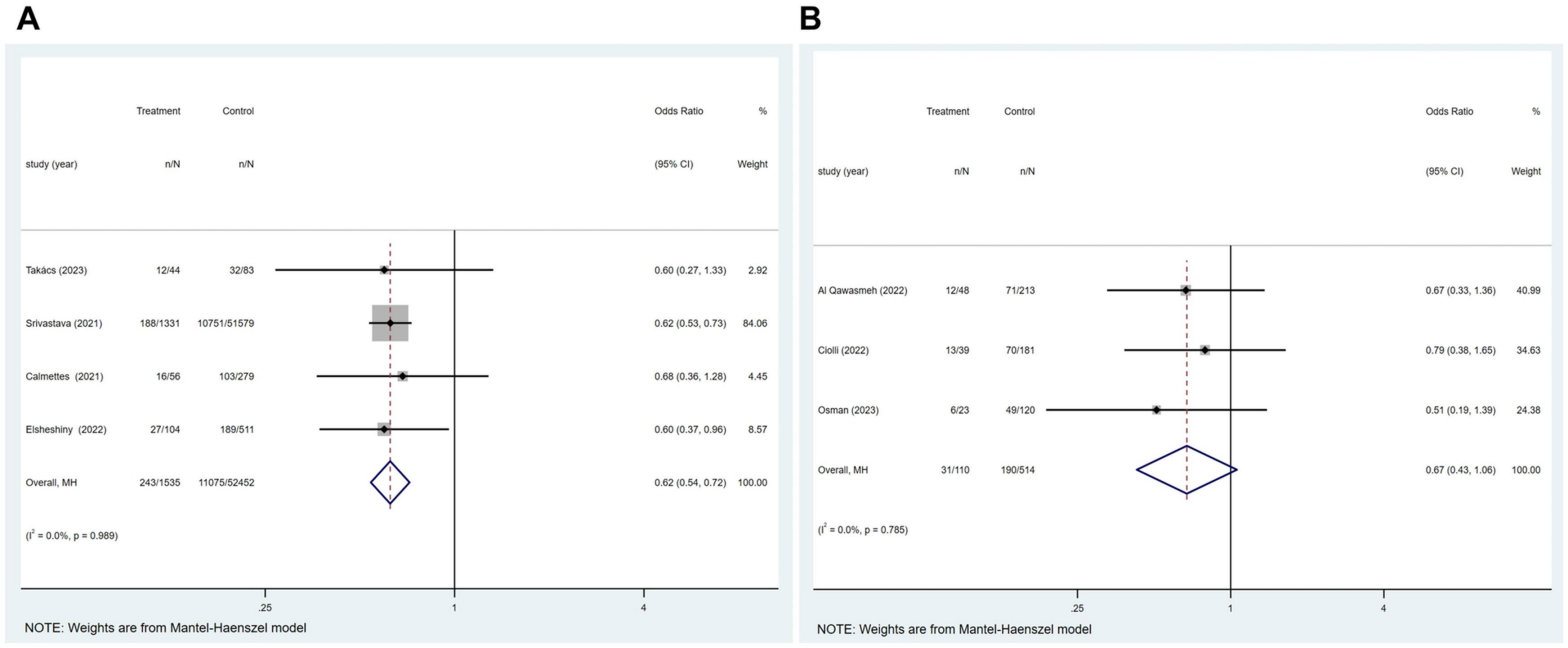
Forest plot illustrating the relationship between COVID-19 and mRS ≤ 2 at different states in patients with ischemic stroke, including **(A)** at hospital discharge; **(B)** 3 months after the onset of ischemic stroke.

Of the 21 studies, four reported an association between COVID-19 and mRS ≤ 2 at hospital discharge in patients with ischemic stroke. Figure 5A suggested that COVID-19 is a contributing factor for a poor mRS score at hospital discharge in patients with ischemic stroke (*p* = 0.989; *I*^2^ = 0.0%; fixed-effects model) (OR, 0.62; 95% CI, 0.54–0.72; *p* < 0.001).

#### 3.6.2 At 3 months after the onset of ischemic stroke

Of the 21 studies, three reported an association between COVID-19 and mRS ≤ 2 at 3 months after the onset of ischemic stroke in patients with ischemic stroke. Figure 5B showed no significant relationship between these two factors (*p* = 0.785; *I*^2^ = 0.0%; fixed-effects model) (OR, 0.67; 95% CI, 0.43–1.06; *p* = 0.086).

### 3.7 Effect of COVID-19 on the length of hospital stay in patients with ischemic stroke

**Figure 6:**
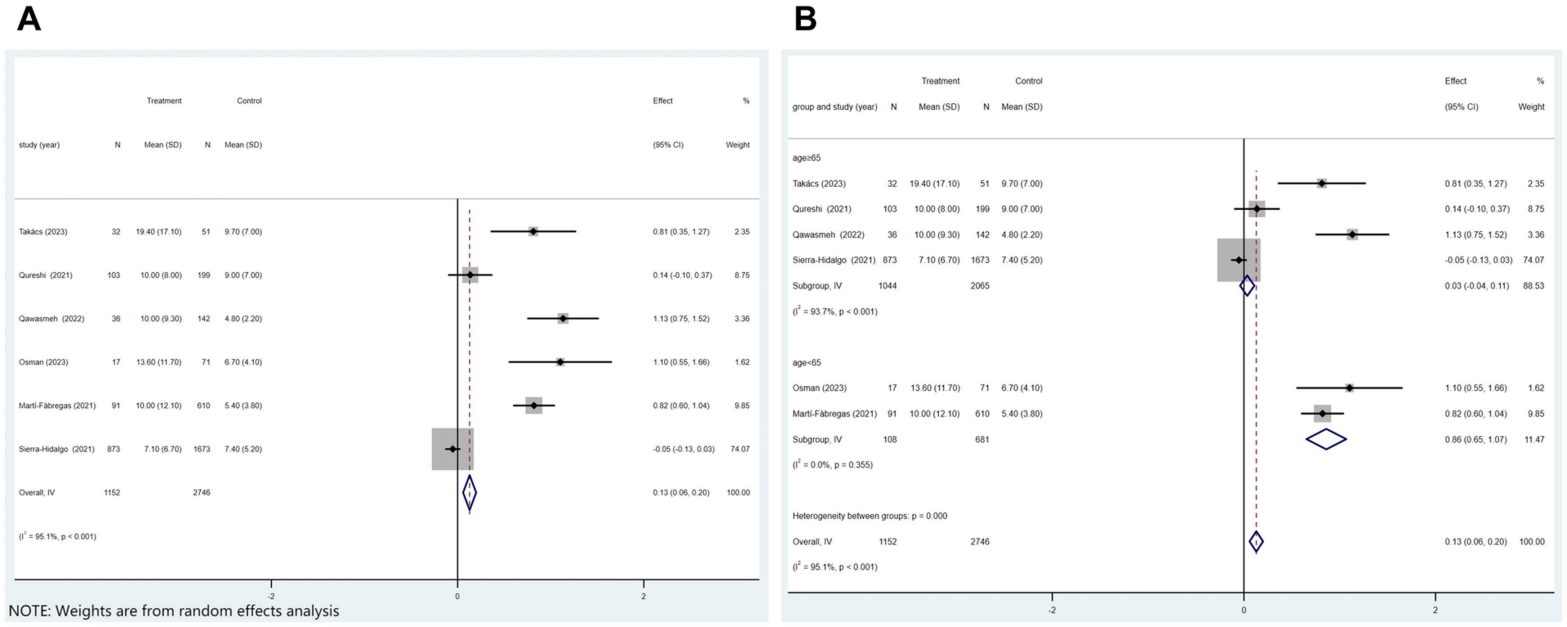
Forest plot illustrating the effect of COVID-19 on the length of hospital stay in patients with ischemic stroke **(A)** and subgroup analyses **(B)**.

Of the 21 studies, six analyzed whether COVID-19 affects the length of hospital stay in patients with ischemic stroke. Figure 6A displayed that patients with COVID-19 ischemic stroke had longer hospital stays (*p* < 0.001 and *I*^2^ = 95.1%; random-effects model) (OR, 0.13; 95% CI, 0.06–0.20; *p* < 0.001) than those without COVID-19. Subgroup analysis was performed according to age (age≥65 vs. age<65). The results showed that COVID-19 patients with ischemic stroke aged≥65 were hospitalized 0.03 days longer than those without COVID-19. COVID-19 patients with ischemic stroke aged<65 were hospitalized for 0.86 days longer than those without COVID-19 (Figure 6B).

### 3.8 Relationship between COVID-19 and the TOAST typing of ischemic stroke

Of the 21 studies, 11 reported a link between COVID-19 and ischemic stroke TOAST typing.

#### 3.8.1 Large-artery atherosclerosis

**Figure 7:**
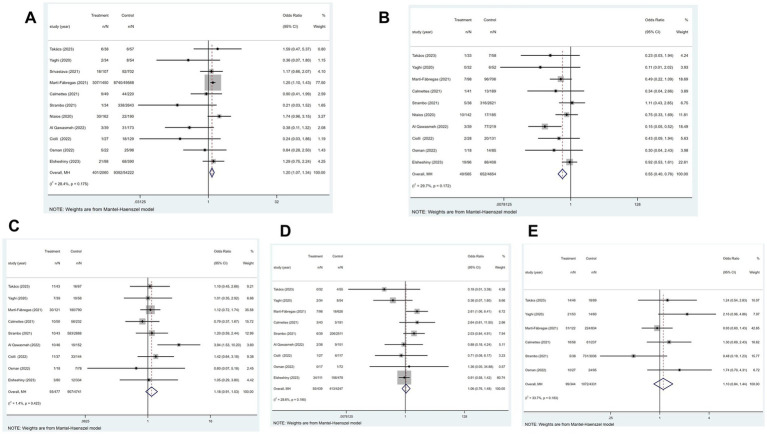
Forest plot illustrating the relationship between COVID-19 and the TOAST typing of ischemic stroke, including **(A)** large-artery atherosclerosis, **(B)** small-vessel disease, **(C)** cardioembolic, **(D)** other determined etiology, and **(E)** cryptogenic phenotype.

Of the 21 studies, 11 reported the large-artery atherosclerosis phenotype of ischemic stroke. Figure 7A showed that patients with COVID-19 and ischemic stroke were more inclined to have a large-artery atherosclerosis phenotype than were patients without COVID-19 but with ischemic stroke (*p* = 0.175; *I*^2^ = 28.4%; fixed-effects model) (OR, 1.20; 95% CI, 1.07–1.34; *p* = 0.002).

#### 3.8.2 Small-vessel disease

Of the 21 studies, 11 reported the small-vessel disease phenotype of ischemic stroke. Figure 7B indicated that patients with ischemic stroke and COVID-19 had a higher risk of developing the small-vessel disease phenotype than those without COVID-19 (*p* = 0.172; *I*^2^ = 29.7%; fixed-effects model) (OR, 0.55; 95% CI, 0.40–0.76; *p* < 0.001).

#### 3.8.3 Cardioembolic phenotype

Of the 21 studies, 11 established the cardioembolic phenotype of ischemic stroke. According to Figure 7C, patients with ischemic stroke with or without COVID-19 did not differ in the prevalence of cardioembolic phenotype (*p* = 0.423; *I*^2^ = 1.4%; fixed-effects model) (OR, 1.18; 95% CI, 0.91–1.53; *p* = 0.212).

#### 3.8.4 Other determined etiology

Of the 21 studies, 11 reported other determined etiology of ischemic stroke. Figure 7D showed that patients with ischemic stroke with or without COVID-19 did not substantially differ in the prevalence of other determined etiology (*p* = 0.190; *I*^2^ = 28.4%; fixed-effects model) (OR, 1.06; 95% CI, 0.76–1.48; *p* = 0.734).

#### 3.8.5 Cryptogenic phenotype

Of the 21 studies, 11 reported the cryptogenic phenotype of ischemic stroke. The heterogeneity test showed significant heterogeneity among the studies (*p* = 0.001; *I*^2^ = 73.0%). Two studies (10, 11) with high heterogeneity were excluded by performing a sensitivity analysis. Using a fixed-effects model, Figure 7E showed that the frequency of cryptogenic phenotype did not importantly change between patients with ischemic stroke with or without COVID-19 (OR, 1.10; 95% CI, 0.84–1.44; *p* = 0.479).

### 3.9 Risk factors for the occurrence of ischemic stroke in patients with COVID-19

Of the 21 studies, eight that included 80,656 patients in total reported the risk factors for ischemic stroke in patients with COVID-19.

#### 3.9.1 Sex

**Figure 8:**
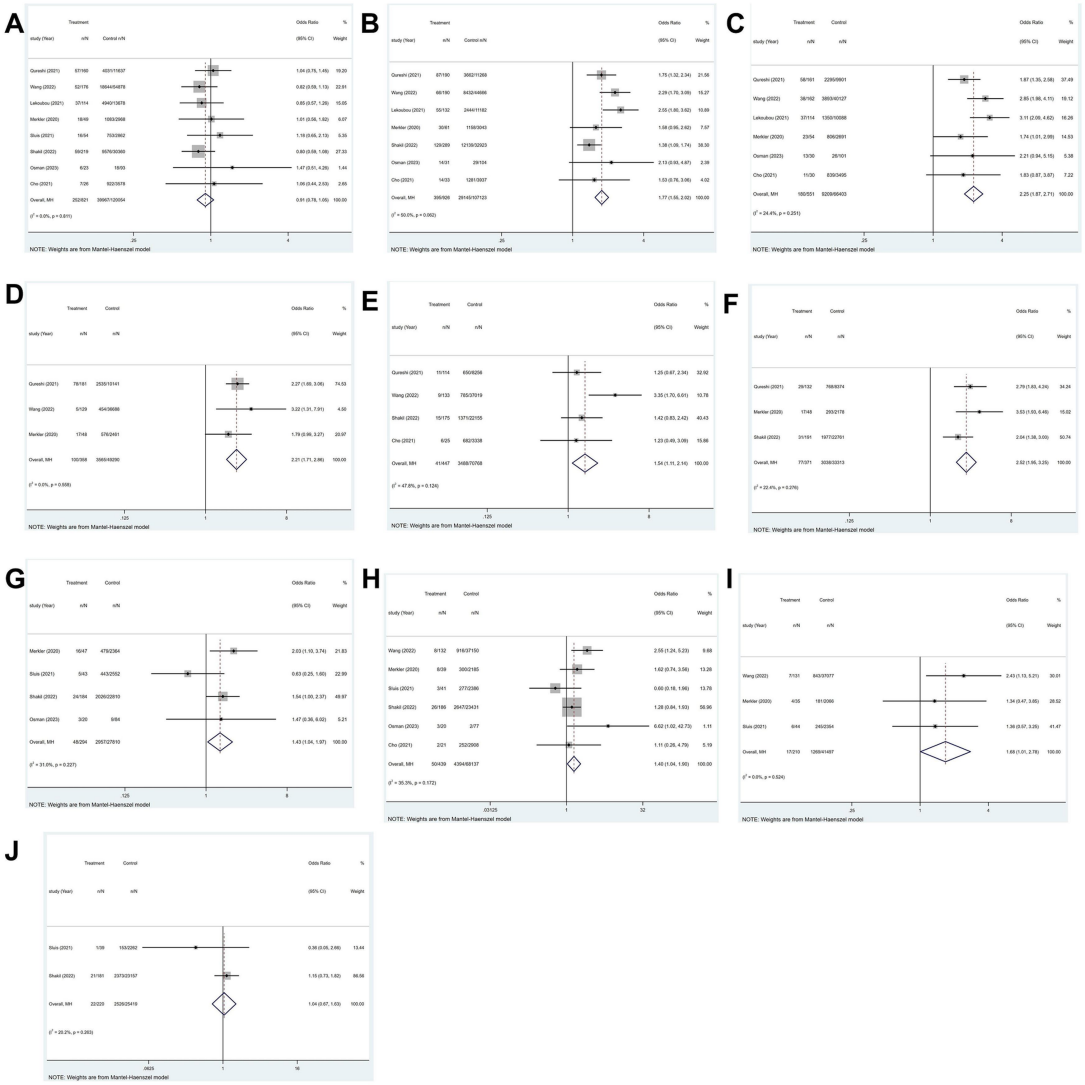
Forest plot illustrating risk factors for ischemic stroke in patients with COVID-19, including **(A)** sex, **(B)** hypertension, **(C)** diabetes, **(D)** hyperlipidemia, **(E)** smoking, **(F)** atrial fibrillation, **(G)** coronary artery disease, **(H)** chronic kidney disease, **(I)** chronic obstructive pulmonary disease, and **(J)** heart failure.

COVID-19 was identified in 569 ischemic stroke patients, 252 of whom were female. In contrast, 39,967 of the 8,087 COVID-19 patients not suffering an ischemic stroke were female. The findings indicated that patients with COVID-19 had no statistically relevant association between sex and ischemic stroke incidence (*p* = 0.811; *I*^2^ = 0.0%; fixed-effects model) (OR, 0.91; 95% CI, 0.78–1.05; *p* = 0.204) (Figure 8A).

#### 3.9.2 Hypertension

All eight studies explored hypertension. The heterogeneity test revealed significant variability among the studies (*p* = 0.008; *I*^2^ = 63.4%). The study (12) with high heterogeneity was excluded through a sensitivity analysis. Utilizing a fixed-effects model, the findings illustrated that hypertension increases likelihood of ischemic stroke in patients with COVID-19 (OR, 1.77; 95% CI, 1.55–2.02; *p* < 0.001)(Figure 8B).

#### 3.9.3 Diabetes

All eight studies explored diabetes. The heterogeneity test indicated significant variability among the studies (*p* = 0.001; *I*^2^ = 70.6%). Two studies (12, 13) exhibited high heterogeneity were excluded through a sensitivity analysis. Employing a fixed-effects model, the findings demonstrated a strong relationship between diabetes and the occurrence of ischemic stroke in patients with COVID-19 (OR, 2.25; 95% CI, 1.87–2.71; *p* < 0.001) (Figure 8C).

#### 3.9.4 Hyperlipidemia

Four of the eight studies investigated hyperlipidemia. The heterogeneity test indicated significant variability among the studies (*p* = 0.063; *I*^2^ = 58.8%). A sensitivity analysis was conducted to exclude the study (12) that exhibited high heterogeneity. Employing a fixed-effects model, results demonstrated that a significant correlation was observed between hyperlipidemia and the occurrence of ischemic stroke among patients with COVID-19 (OR, 2.21; 95% CI, 1.71–2.86; *p* < 0.001) (Figure 8D).

#### 3.9.5 Smoking

Four of the eight studies investigated smoking. Findings indicated that among patients with COVID-19, smoking was substantially linked to the risk of ischemic stroke (*p* = 0.124; *I*^2^ = 47.8%; fixed-effects model) (OR, 1.54; 95% CI, 1.11–2.14; *p* = 0.01) (Figure 8E).

#### 3.9.6 Atrial fibrillation

Four of the eight studies investigated atrial fibrillation (AF). The heterogeneity test revealed significant variability among the studies (*p* = 0.067; *I*^2^ = 58.0%). A sensitivity analysis was performed to exclude the study (12) that demonstrated high heterogeneity. Utilizing a fixed-effects model, the results indicated that atrial fibrillation exerts a significant influence on the prevalence of ischemic stroke in patients with COVID-19 (OR, 2.52; 95% CI, 1.95–3.25; *p* < 0.001) (Figure 8F).

#### 3.9.7 Coronary artery disease

Four of the eight studies explored coronary artery disease. The results showed that among COVID-19 patients, coronary artery disease and the incidence of ischemic stroke were significantly correlated (*p* = 0.227; *I*^2^ = 31.0%; fixed-effects model) (OR, 1.43; 95% CI, 1.04–1.97; *p* = 0.027) (Figure 8G).

#### 3.9.8 Chronic kidney disease

Six of the eight studies reported on chronic kidney disease. The outcomes demonstrated a significant relationship between the occurrence of ischemic stroke and chronic kidney disease in patients with COVID-19 (*p* = 0.172; *I*^2^ = 35.3%; fixed-effects model) (OR, 1.40; 95% CI, 1.04–1.90; *p* = 0.028) (Figure 8H).

#### 3.9.9 Chronic obstructive pulmonary disease

Three of the eight studies investigated chronic kidney disease. According to the findings, there was a strong link between chronic obstructive pulmonary disease (COPD) and ischemic stroke in patients with COVID-19 (*p* = 0.524; *I*^2^ = 0.00%; fixed-effects model) (OR, 1.68; 95% CI, 1.01–2.78; *p* = 0.047) (Figure 8I).

#### 3.9.10 Heart failure

Three of the eight studies reported on heart failure. The heterogeneity test demonstrated significant variability among the studies (*p* = 0.008; *I*^2^ = 79.5%). A sensitivity analysis was performed to exclude the study (14) that demonstrated high heterogeneity. A fixed-effects model was utilized, as the results show, heart failure does not elevate the risk of ischemic stroke in patients with COVID-19 (OR, 1.04; 95% CI, 0.67–1.63; *p* = 0.850) (Figure 8J).

### 3.10 Sensitivity analysis and risk factor analysis

Incidence of ischemic stroke in patients with COVID-19, effect of COVID-19 on in-hospital mortality in patients with ischemic stroke and effect of COVID-19 on the length of hospital stay in patients with ischemic stroke have highly heterogeneity. Subgroup analyses were performed separately to account for the highly heterogeneous incidence of ischemic stroke among patients with COVID-19 and the effect of COVID-19 on the length of stay among patients with ischemic stroke (Figures 2B, 6B).

We addressed the significant heterogeneity in in-hospital mortality by age stratification (age≥65 vs. age<65) and sample size (large sample *n* ≥ 1,000 vs. small sample *n* < 1,000), but the heterogeneity still existed in the age subgroup (age≥65 group *I*^2^ = 76.36%, *p* = 0.000; age <65 group, *I*^2^ = 91.74%, *p* = 0.000) and in the sample size subgroup (large sample *I*^2^ = 90.25%, *p* = 0.000; small sample *I*^2^ = 84.68%, *p* = 0.000).

### 3.11 Publication bias

**Figure 9:**
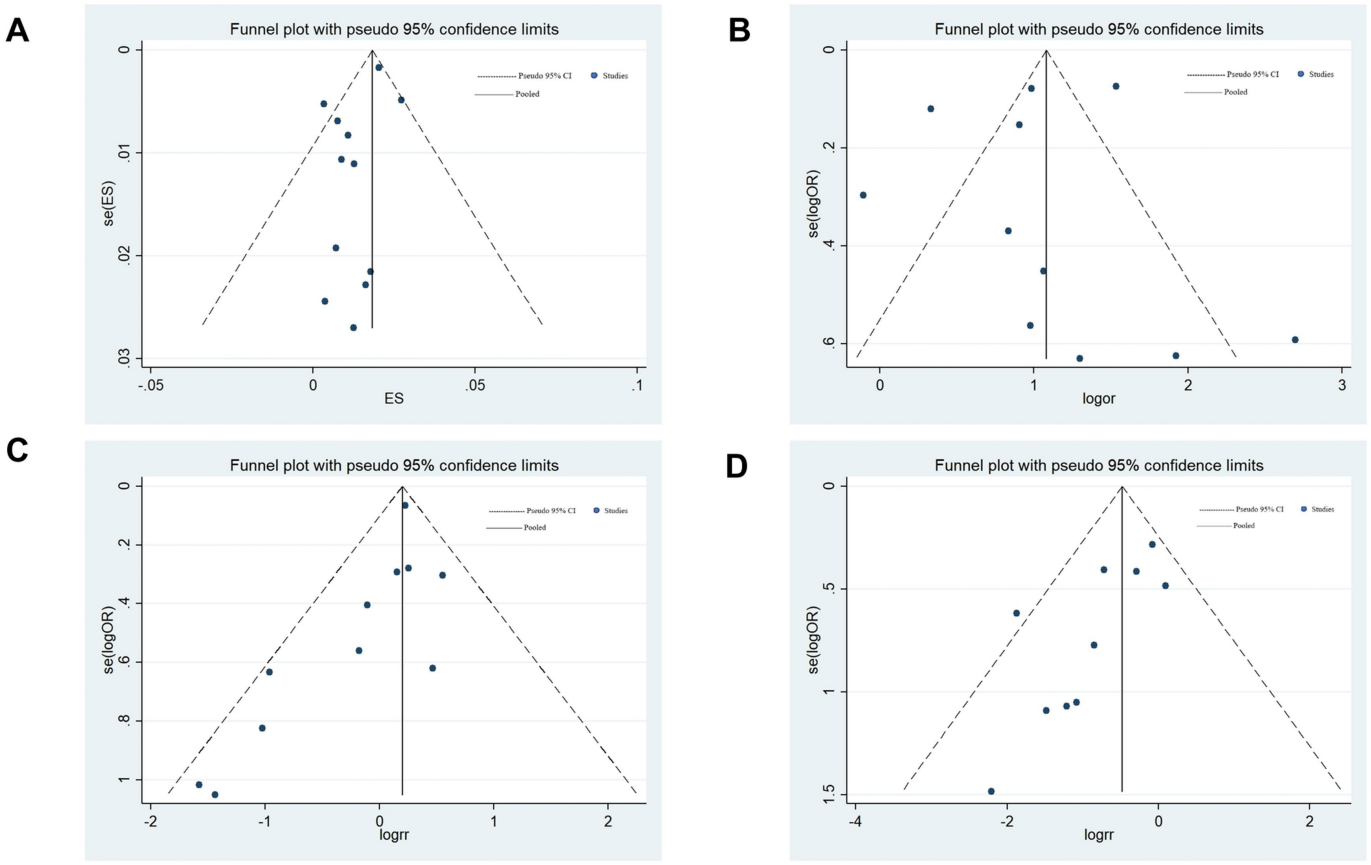
Begg’s funnel plots. **(A)** Relationship between COVID-19 and the incidence of ischemic stroke. **(B)** Relationship between COVID-19 and in-hospital mortality in patients with ischemic stroke. **(C)** Relationship between COVID-19 and the incidence of the large artery atherosclerosis phenotype of ischemic stroke. **(D)** Relationship between COVID-19 and the incidence of the small vessel disease phenotype of ischemic stroke.

Begg’s funnel plot and Egger’s regression test were employed to evaluate publication bias in the meta-analyses. The visually asymmetric relationship between COVID-19 and the incidence of ischemic stroke was demonstrated by Begg’s funnel plot (Figure 9A); however, Egger’s regression test (*p* = 0.113) revealed no substantial publication bias. Begg’s funnel plot was visually asymmetric (Figure 9B), which depicted the link between COVID-19 and in-hospital mortality in patients with ischemic stroke; however, Egger’s regression test (*p* = 0.699) showed no substantial publication bias.

Regarding the association between COVID-19 and the incidence of the large-artery atherosclerosis phenotype of ischemic stroke, both Begg’s funnel plot (asymmetrical; Figure 9C) and Egger’s regression test (*p* = 0.035) revealed publication bias. This bias may stem from the fact that included studies were more likely to publish studies that found significant associations or that different studies controlled for confounding factors to different degrees. Afterward, publication bias was assessed through a trim-and-fill analysis, as previously delineated (15). The pooled OR for large artery atherosclerosis subtype was 1.20 (95%CI: 1.07–1.34) to 1.22 (1.09–1.37), with a change of only 1.7% (adjusted *p* = 0.001), indicating that publication bias did not significantly affect the pooled results and our results are reliable.

Egger’s regression test (*p* = 0.021) and Begg’s funnel plot (asymmetrical; Figure 9D) of the association between COVID-19 and the incidence of the small-vessel disease phenotype of ischemic stroke both showed publication bias. This bias may mainly result from missing studies with a small sample size or selectively reporting positive results in the included studies. Trim-and-fill analysis revealed that the pooled OR for small-vessel disease phenotype of ischemic stroke was 0.62 (0.447–0.86) from 0.55 (95%CI: 0.40–0.76) in the trimmed and fill analyses. The adjusted OR increased by 7%, suggesting that the original results may overestimate the protective effect by 12.7%, but the OR was less than 1.0, *p* = 0.004, indicating that publication bias did not significantly affect the pooled results, and our results are credible.

## 4 Discussion

Attention is gradually being drawn to the part COVID-19 plays in the incidence of cerebrovascular disease. Current studies suggest that COVID-19 may have some influence on the morbidity, mortality, and prognosis of ischemic stroke (4, 16–19); however, the evidence is not conclusive. In addition, in patients with COVID-19, hypertension, diabetes, dyslipidemia, coronary artery disease, and obesity raise the probability of ischemic stroke (20, 21). In this systematic review, the correlation between the occurrence of ischemic stroke in patients with COVID-19 and AF, chronic kidney disease, COPD, or heart failure was analyzed. Twenty-one studies with quality assurance and credible results were included.

After analyzing 21 studies, compared to those without COVID-19, patients with COVID-19 were at increased likelihood of developing an ischemic stroke. Based on certain research, patients with COVID-19 have a 1–1.5% chance of having an ischemic stroke (5, 14), which is consistent with our results. In addition, we found that the incidence of patients aged≥65 was higher than that of patients aged<65. This may be related to the differences in the burden of comorbidities and clinical management between the two groups. Our study revealed that the in-hospital mortality rate in patients with COVID-19 and ischemic stroke was markedly elevated in comparison to patients with ischemic stroke but without COVID-19 (10, 13, 14, 22–29). In addition, the mortality rate in patients with ischemic stroke at 3 months after the onset of ischemic stroke was significantly higher than that in patients with ischemic stroke but without COVID-19 (11, 29–31), suggesting that there is an elevated risk of in-hospital mortality in patients with ischemic stroke after a 3-month onset period, with the potential for the presence of the COVID-19 to be a contributing factor. These outcomes align with those of previous research studies (20, 32). Patients with COVID-19 experience respiratory distress, multi-organ failure, elevated levels of serum inflammatory markers, and fibrin activation, which increases incidence and mortality rates in those with ischemic stroke (20, 21, 33, 34). Patients with COVID-19 and ischemic stroke are discharged with a poor functional status, suggesting that a major factor in deciding the prognosis of these patients is COVID-19. In contrast, following 3 months of illness, there was little variation in the mRS score between patients with COVID-19 and ischemic stroke and those without COVID-19 but with ischemic stroke. Patients who have both COVID-19 and an ischemic stroke are more likely to have poor outcomes since COVID-19 affects nearly all organ systems (35). In our study, patients with ischemic stroke and COVID-19 stayed 0.13 days longer in the hospital than did patients without COVID-19. This can be attributed to the fact that patients with COVID-19 and ischemic stroke have a poor overall clinical status. In addition, we found that the hospital stays of the group aged < 65 was longer than that of the group aged ≥ 65. This may be related to the differences in clinical decision-making and medical resource allocation between the two groups. Compared to previous studies (36, 37), the length of hospital stay observed in our study was lower. This discrepancy can be ascribed to the varying severity of patients’ conditions when hospitalized. The incidence of large-artery atherosclerosis and small-vessel disease phenotype of ischemic stroke was higher in patients with COVID-19 and ischemic stroke compared to those without COVID-19. However, there was little variation in the incidence of cardioembolic, other determined etiologies and cryptogenic phenotype. Common risk factors for the onset of ischemic stroke in patients with COVID-19 were hypertension, diabetes, smoking, obesity, dyslipidemia, chronic renal disease, coronary artery disease, and COPD, with no significant correlation with age. Although many COVID-19 comorbidities, such as myocardial infarction, heart failure, and chronic kidney disease, may increase the occurrence of ischemic stroke (38–41), the primary risk factors remain those typically associated with ischemic stroke.

The mechanism of action of COVID-19 in ischemic stroke remains unclear. Our study suggests several possible mechanisms. In 20–55% of hospitalized patients with COVID-19, elevated D-dimer levels of more than twice the normal value, a slightly prolonged prothrombin time (1–3 s above normal), and mild thrombocytopenia have been observed (42). These changes can lead to the development of sepsis-induced coagulation disorder, which is a precursor for disseminated intravascular coagulation (43), which in turn can increase the risk of thrombosis and ischemic stroke. In addition, antiphospholipid antibodies are present in severely ill patients with COVID-19 and multiple cerebral infarctions. These antibodies can increase the risk of thrombosis by promoting cellular activation, inhibiting the natural anticoagulant and fibrinolytic systems, and triggering the complement system (44). Lung cells in the alveolar lining express angiotensin-converting enzyme 2 (ACE2) receptors, which SARS-CoV-2 uses to infect the host and inflict lung injury. In addition to the lungs, endothelial cells also have high levels of expression for ACE2 receptors (45), and the infection of these cells can elicit an inflammatory response (46), which is considered one of the precursors for COVID-19-related thrombotic complication. An essential function of ACE2 is to control the renin-angiotensin system (47), and ACE2-induced angiotensin 1–7 production promotes angiogenesis, oxidative stress inhibition, and neuroinflammatory resistance in brain tissues and improves cerebral blood flow (48). Through the induction of receptor endocytosis, SARS-CoV-2 depletes ACE2 (43). This process causes endothelial dysfunction in the heart and brain, which raises sympathetic activity, impairs blood pressure autoregulation, causes vasoconstriction, and ultimately causes ischemia in the affected organs (49). Viral infection causes sustained uncontrolled activation of the immune system with an over-release of cytokines (50), resulting in a cytokine storm that leads to systemic symptoms, systemic inflammation, and multi-organ dysfunction, and, if not properly treated, multi-organ failure and coagulation disorders (51). SARS-CoV-2 directly invades vascular endothelial cells through angiotensin-converting enzyme 2 (ACE2) receptor, triggering the continuous activation of the NF-κB signaling pathway (52, 53), leading to the massive release of pro-inflammatory cytokines such as IL-6 and TNF-*α*. This “cytokine storm” not only aggravates the inflammatory infiltration in the plaque, promotes the activation of macrophages and the secretion of matrix metalloproteinases (MMP-2/9), but also causes the detachment of endothelial glycocalyx, exposing the procoagulant surface of the vessel wall and accelerating platelet adhesion. Second, virus-induced monocyte activation promotes the overexpression of tissue factor (TF), which initiates the exogenous coagulation pathway. Meanwhile, extracellular traps (NETs) released by neutrophils provide scaffolds for thrombus formation and enhance fibrin deposition (54, 55). Together, these changes lead to large-artery atherosclerosis phenotype of ischemic stroke. After virus invasion of brain microvascular endothelial cells, the activation of MMP-9 mediated by Spike protein leads to the degradation of tight junction proteins and increases the permeability of the blood–brain barrier (56). At the same time, excessive activation of the complement system, especially the C5a-C5aR1 axis, drives platelet microaggregation and fibrin deposition in small vessels (57). In addition, hypoxia-induced up-regulation of HIF-1α further aggravates vasogenic edema and forms a vicious cycle of “inflammation and hypoxia” (58, 59). SARS-CoV-2 induces the small-vessel disease phenotype of ischemic stroke by causing the destruction of the blood–brain barrier and microcirculation disorders through this unique mechanism. The dominant distribution of large-artery atherosclerosis phenotype of ischemic stroke and small-vessel disease phenotype of ischemic stroke in patients with COVID-19 reflects the specific targeting of the virus to the vascular system, as compared with other TOAST subtypes such as cardioembolic phenotype of ischemic stroke. Cardioembolic phenotype of ischemic stroke usually requires structural heart disease, and although COVID-19 can induce atrial fibrillation, its incidence is relatively low (about 6–8%) and most of them are transient (60). In contrast, direct viral damage to the vascular endothelium and systemic inflammatory responses are more prevalent and persistent. Coagulation function tests showed that patients with COVID-19 presented with a special “bipolar” coagulation disorder, which manifested as a hypercoagulable state with significant increases in D-dimer and fibrinogen in the early stage, and could develop into consumption coagulopathy in the later stage. This dynamic change allows the risk of arterial thrombosis *in situ* to persist, while embolic events are relatively rare (61).

The results of this study can be used to guide clinical practice, particularly with regard to risk stratification and treatment strategies. A more accurate understanding of the impact of early COVID-19 on clinical outcomes in patients with ischemic stroke may provide insights for future prevention and treatment of ischemic stroke associated with the virus pandemic. In the management of COVID-19 patients, intensive ischemic stroke surveillance programmes should be implemented for groups of patients with specific risk factors, who should receive more intensive stroke screening and preventive anticoagulant therapy as appropriate for their condition. This high-risk group mainly includes three categories: first, patients with traditional vascular risk factors including hypertension, diabetes, hyperlipidemia and smoking history; Patients with cardiovascular disease, such as atrial fibrillation, coronary artery disease and chronic kidney disease; Third, patients with respiratory complications, especially chronic obstructive pulmonary disease. For hospitalized patients, daily systemic neurological evaluations are recommended by clinicians. When patients develop new neurological symptoms, head imaging should be initiated immediately. It is worth noting that for patients with severe disease requiring mechanical ventilation or patients with significant elevated inflammatory markers, such as D-dimer and CRP, it is still necessary to be highly vigilant against thromboembolic events even if traditional risk factors are lacking, and it is recommended to include them in the scope of intensive monitoring. In terms of anti-thrombotic treatment strategy, for patients without atrial fibrillation, if there is evidence of hypercoagulation, such as elevated D-dimer, low molecular weight heparin prophylactic anticoagulation can be considered. For patients with atrial fibrillation, it is necessary to ensure the continuity of anticoagulant therapy, prioritize direct oral anticoagulants, and closely monitor coagulation function. It is suggested that clinicians should adjust the prevention and treatment strategies in time according to the individual characteristics of patients and the dynamic monitoring results.

A limitation of our study is the high degree of heterogeneity in the results of most analyses. we conducted subgroup analyses based on age for the incidence of COVID-19 combined with ischemic stroke and hospital length of stay. For the high heterogeneity of in-hospital mortality in COVID-19 patients with ischemic stroke, we conducted subgroup analysis by age and sample size, but the heterogeneity still persisted. This suggests that, in addition to differences in demographic characteristics and study sizes, there may be key confounders that have not been quantified. On the basis of the pathophysiology of COVID-19, we speculate that COVID-19 severity may be a central source of heterogeneity. However, due to the general lack of uniform grading criteria in the included literature, this study could not be quantitatively combined for analysis. In addition, when analyzing risk factors, our study had limitations. First, we verified the effects of age on the effect size of each risk factor through meta-regression analysis, and the results showed that age may not be a significant confounding factor in this study. However, other potentially confounding variables, particularly disease severity for COVID-19, were not included in the analysis due to the lack of uniform standard reporting in the original study and the small sample size. This lack of data may lead to residual confounding bias, as severely ill patients tend to have both a higher underlying disease burden and poorer clinical outcomes. Second, our study found that hypertension, diabetes, hyperlipidemia, smoking, atrial fibrillation, coronary artery disease, and chronic kidney disease were independently associated with adverse COVID-19 outcomes. It should be emphasized, however, that these factors may have complex interactions. Although we attempted to explore this interaction through subgroup analysis, we were limited by the lack of interaction item reporting in the original data and were unable to draw definitive conclusions. Future large-scale collaborative studies are needed to systematically assess the combined effects of combinations of risk factors to guide precise treatment strategies. Only 3 studies reported the association between COVID-19 and mRS ≤ 2 in patients with ischemic stroke at 3 months after the onset of ischemic stroke. Although the observed trend in effect (odds ratio, 0.67; *p* = 0.086) suggested that COVID-19 infection may reduce the odds of a good functional recovery, the result lacked significance. This may be due to the included sample size *N* = 403, which may be too small to detect a true effect rather than a true no association. Further studies with larger sample sizes are needed to confirm this finding. In the included literature, only two studies (22, 26) stratified the outcome of ischemic stroke based on the severity of COVID-19 infection. However, these two studies have obvious limitations. First, the sample size was small and the statistical power was insufficient. Second, the criteria and levels of stratification of the severity of COVID-19 infection that were used in the two studies were significantly different. Because of these methodological heterogeneity, we were not able to perform further meta-analysis. This missing data limit the systematic assessment of the potential effect of the severity of COVID-19 infection on the incidence, mortality, and clinical outcomes of ischemic stroke. Of note, despite these limitations, the results of both these studies showed an association between severe COVID-19 infection and worse stroke outcomes. This finding suggests that the severity of COVID-19 infection may be an important factor in the outcome of ischemic stroke, but more studies with uniform standards and large samples are needed to confirm this finding. To analyze the relationship of COVID-19 with the incidence of ischemic stroke, in-hospital mortality in patients with ischemic stroke, incidence of large-artery atherosclerosis phenotype and small-vessel disease phenotype ischemic stroke, the number of articles included was≥10, and a publication bias analysis was conducted. The remaining analyses each had less than 10 articles, which may have led to biased results. Nevertheless, the results yielded by the meta-analysis were consistently positive and representative. This article analyzed the impact of COVID-19 on mortality and adverse outcomes after 3 months of ischemic stroke onset. However, patients with COVID-19 combined with ischemic stroke may be at risk of long-term adverse outcomes, such as recurrent ischemic stroke or bleeding. Further studies are needed to determine the long-term effects of COVID-19 on ischemic stroke patients and the clinical therapeutic interventions for these patients. Language limitation is a limitation of this study, which may introduce language bias and exclude relevant studies from non-English speaking regions. However, our initial search revealed that most of the core scientific results in the field of COVID-19 and ischemic stroke prognosis have been published in English-language journals. Nonetheless, we recognize that some geographically specific findings may have been overlooked because they were published in local journals, and the risk of publication bias for negative results may be amplified. We look forward to more literature on language systems in this area in the future to ensure a more comprehensive study. Additionally, as COVID-19 is a disease that has only emerged recently, findings regarding its effect on ischemic stroke may still be considered preliminary. As the body of literature exploring the relationship between COVID-19 and ischemic stroke continues to expand, there is a necessity for meta-analyses that encompass a wider range of studies and larger sample sizes.

## 5 Conclusion

COVID-19 was linked to the incidence, mortality, and prognosis of ischemic stroke. Additionally, COVID-19 correlated with an elevated frequency of large-artery atherosclerosis, small-vessel disease, and cardioembolic phenotype of ischemic stroke. Furthermore, patients with COVID-19 were more susceptible to ischemic stroke due to a history of smoking, hypertension, diabetes, hyperlipidemia, AF, chronic kidney disease, coronary artery disease, and COPD. This will offer new insights and ideas for the treating and preventing of ischemic stroke in the setting of COVID-19.

## Data Availability

The original contributions presented in the study are included in the article/Supplementary material, further inquiries can be directed to the corresponding author.
