# Supplementary material for: Effects of coronavirus disease 2019 on the incidence, mortality, and prognosis of ischemic stroke: a systematic review and meta-analysis
Source: Front Neurol. 2025 May 13;16:1486887. doi: 10.3389/fneur.2025.1486887 (PMC12106046; doi:10.3389/fneur.2025.1486887)
Supplement: Supplementary file 1 [file Table_1.docx]

**predefined keywords:**

“Ischemic Stroke OR Ischemic Strokes OR Stroke, Ischemic OR Ischaemic Stroke OR Ischaemic Strokes OR Stroke, Ischaemic OR Cryptogenic Ischemic Stroke OR Cryptogenic Ischemic Strokes OR Ischemic Stroke, Cryptogenic OR Stroke, Cryptogenic Ischemic OR Cryptogenic Stroke OR Cryptogenic Strokes OR Stroke, Cryptogenic OR Cryptogenic Embolism Stroke OR Cryptogenic Embolism Strokes OR Embolism Stroke, Cryptogenic OR Stroke, Cryptogenic Embolism OR Wake-up Stroke OR Stroke, Wake-up OR Wake up Stroke OR Wake-up Strokes OR Acute Ischemic Stroke OR Acute Ischemic Strokes OR Ischemic Stroke, Acute OR Stroke, Acute Ischemic OR brain infarction OR acute cerebral infarction OR cerebral infarct OR cerebral ischemia OR Cerebral infarction OR Cerebral Infarctions OR Infarctions, Cerebral OR Infarction, Cerebral OR Cerebral Infarct OR Cerebral Infarcts OR Infarct, Cerebral OR Infarcts, Cerebral OR Cerebral Infarction, Left Hemisphere OR Left Hemisphere, Infarction, Cerebral OR Infarction, Left Hemisphere, Cerebral OR Left Hemisphere, Cerebral Infarction OR Cerebral, Left Hemisphere, Infarction OR Infarction, Cerebral, Left Hemisphere OR Subcortical Infarction OR Infarction, Subcortical OR Infarctions, Subcortical OR Subcortical Infarctions OR Posterior Choroidal Artery Infarction OR Anterior Choroidal Artery Infarction OR Cerebral Infarction, Right Hemisphere OR Right Hemisphere, Cerebral Infarction OR Infarction, Right Hemisphere, Cerebral OR Right Hemisphere, Infarction, Cerebral OR Cerebral, Right Hemisphere, Infarction OR Infarction, Cerebral, Right Hemisphere OR Brain Ischemias OR Ischemia, Brain OR Ischemic Encephalopathy OR Encephalopathy, Ischemic OR Ischemic Encephalopathies OR Cerebral Ischemia OR Cerebral Ischemias OR Ischemias, Cerebral OR Ischemia, Cerebral” AND “SARS-CoV-2 OR SARS-CoV-2 Virus OR SARS CoV2 Virus OR SARS-CoV-2 Viruses OR Virus, SARS-CoV-2 OR 2019 Novel Coronavirus OR 2019 Novel Coronaviruses OR Coronavirus, 2019 Novel OR Novel Coronavirus, 2019 OR COVID-19 Virus OR COVID 19 Virus OR COVID-19 Viruses OR Virus, COVID-19 OR Wuhan Coronavirus OR Coronavirus, Wuhan OR COVID-19 Virus OR COVID-19 Viruses OR Virus, COVID-19 OR Viruses, COVID-19 OR Coronavirus Disease 2019 Virus OR Severe Acute Respiratory Syndrome OR Coronavirus 2 OR SARS Coronavirus 2 OR Coronavirus 2, SARS OR 2019-nCoV OR Wuhan Seafood Market Pneumonia Virus Filters”.
